# Menthol induces apoptosis and inhibits proliferation and migration of nonsmall cell lung carcinoma in vitro and in vivo through Akt pathway

**DOI:** 10.1111/crj.13713

**Published:** 2023-11-27

**Authors:** Zhiyu Liu, Chunlin Li, Ling Mu, Haiyang Hu, Xiong Qin

**Affiliations:** ^1^ Department of Critical Care Medicine, Shanghai Tenth People's Hospital Tongji University School of Medicine Shanghai China; ^2^ Department of Orthopaedics, Qilu Hospital of Shandong University, Shandong University Centre for Orthopaedics, Advanced Medical Research Institute, Cheeloo College of Medicine Shandong University Jinnan China; ^3^ Trauma Center, Shanghai General Hospital Shanghai Jiao Tong University School of Medicine Shanghai China; ^4^ Department of Vascular Surgery Shanghai Sixth People's Hospital Affiliated to Shanghai Jiao Tong University School of Medicine Shanghai China; ^5^ Department of Thoracic Surgery, Shanghai Pulmonary Hospital School of Medicine, Tongji University Shanghai China

**Keywords:** Akt signaling pathway, apoptosis, cell motility, menthol, NSCLC

## Abstract

**Background:**

About 40% of nonsmall cell lung cancers (NSCLCs) have already progressed in an advanced stage at the time of diagnosis. Development of effective prevention and therapy approaches against NSCLC is critical for reducing mortality. As a fundamental ingredient of peppermint oil, menthol has been demonstrated to possess an antitumor activity in several types of carcinomas. However, the potential role of menthol on NSCLC has not been reported. The present study aims to investigate the effect and underlying mechanism of menthol on proliferation, apoptosis, and mobility of human lung adenocarcinoma.

**Methods:**

Cell apoptosis was examined by MTT and flow cytometry. The motility of cells was determined by Transwell assay. Western blot analysis was performed to determine expression level of proteins. In vivo model of nude mice was established for evaluating the influence of menthol on tumorigenicity of A549 cells. The expression lentiviral vector of Akt was established in NSCLC cells for further verifying the inhibiting effect of menthol on survival and mobility of NSCLC cells via Akt pathway.

**Results:**

The results showed that menthol promoted A549 cell apoptosis, suppressed cell proliferation, and motility by altering the phosphorylated protein level of Akt. Menthol enhanced the expression level of Bax while decreasing expression of Bcl‐2, Caspase‐3, and MMPs proteins. In vivo experiments suggested that menthol exhibited an inhibitory effect in tumor growth on xenografts. These results were further validated in Akt over‐expressed A549 and H1299 cells.

**Conclusions:**

Menthol could display an inhibitory effect on NSCLC cells through Akt signaling pathway, making it a potential target for NSCLC treatment.

## Graphical abstract

Menthol could display an inhibitory effect on A549 cells through Akt signaling pathway, and menthol might be a potential target for NSCLC treatment.

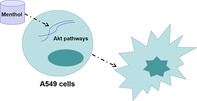

## 1 INTRODUCTION

Lung cancer, of which the most frequently occurring is nonsmall cell lung cancer (NSCLC), still ranks among the most common cancers all around the world.^1^, ^2^ Typically, 40% of NSCLCs have already progressed in an advanced stage at the time of diagnosis when surgery is not applicable. Despite significant advancement in the therapy of early‐stage lung cancer, survival rates for advanced disease remain extremely low.^3^ Therefore, development of effective prevention and therapy approaches against NSCLC is critical for reducing mortality.

As multiple natural herbal extracts have attracted increasing attention because of their anticancer potential,^4^, ^5^ the role of menthol (C_10_H_20_O, molecular weight = 156) captured our attention. As a naturally existing monocyclic terpene alcohol, menthol is purified from peppermint essential oil, which has been widely employed in condiments, pharmaceutical products and cosmetics. It is generally considered safe and has been approved for over‐the‐counter external use.^6^ Menthol has also been proved to have antitumor potential by apoptosis induction and motility inhibition in malignancies such as human leukemia, prostate cancer and colon cancer.^7^, ^8^, ^9^ However, the effect and underlying mechanism of menthol on NSCLC has not been elucidated yet.

Increased activation of the Akt pathway leads to numerous hallmarks of cancer. In NSCLC, this pathway has been greatly correlated to tumorigenesis. Deregulated activation of Akt has been found in nearly 70% cases of NSCLC, so this target has become attractive for novel anticancer therapies.^10^

In the present study, we investigated the possible anticancer activity of menthol on NSCLC cell line A549 in vitro and in vivo, and the possible molecular mechanisms were preliminarily discussed. We demonstrated that menthol could induce apoptosis of A549 cells and suppress cell proliferation and motility while increasing the expression level of Bax and decreasing Bcl‐2, Caspase‐3, and matrix metalloproteinases (MMPs) protein levels. The phosphorylation level of Akt (p‐Akt) changed in the meantime. The data suggested that menthol might inhibit A549 cells through Akt signaling pathway, and menthol might be able to play a role in the treatment of NSCLC.

## 2 MATERIALS AND METHODS

## 3 CELL CULTURE

The human lung adenocarcinoma cell lines A549 and H1299 cells were supplied by the Institute of Biochemistry and Cell Biology (Shanghai Institutes for Biological Sciences, CAS). The cell line was authenticated by short tandem repeat fingerprinting and mycoplasma contamination was tested before use. Cells were cultured in Dulbecco's modified Eagle's medium (DMEM; Life Technologies, Grand Island, NY) with 10% fetal bovine serum (FBS; Life Technologies) supplemented. Cells were prepared at 37°C in a humidified 5% CO_2_ incubator.

### 3.1 MTT assays

Cells were seeded firstly into 96‐well culturing plates at a density of 1 × 10^4^ cells per well. Afterwards, cells were stimulated with indicated concentrations (0, 0.05, 0.1, 0.2, 0.4, and 0.8 mM) of menthol (63 670, Sigma‐Aldrich, Germany) and SC79 (2, 4, and 8 μg/mL) (HY‐18749, MedChemExpress, USA) in medium and incubated for 24 or 48 h. Then the medium was removed, and cells were furtherly incubated with 100 μL MTT (M5655, Sigma‐Aldrich, Germany) adding to each well (0.5 mg/mL final concentration). After 4 h, the medium was removed, and 100 μL DMSO was added. Finally, with 15 min of incubation at 37°C, the absorbance of each well was measured at 570 nm with a Microplate Reader (Thermo Fisher, USA).

### 3.2 Cell apoptosis detection

According to the manufacturer's instructions, cell apoptosis was determined with a FITC‐Annexin V apoptosis detection kit (BD biosciences, USA). Briefly, A549 cells were seeded into 6‐well plates and added with menthol (0.8 mM) and/or SC79 (4 μg/mL) for 24 h. Then cells were resuspended with binding buffer at a 1 × 10^6^ cells/mL concentration. After 5 μL of FITC‐Annexin V and 5 μL of PI were added, cells were incubated in darkness at room temperature for 20 min. Cell apoptosis rate was then measured by flow cytometry (BD FACSCalibur, USA) with 400 μL binding buffer added to each tube.

Intracellular Caspase‐3 activity levels were determined using Caspase‐3 Activity Assay Kit (Beyotime, Jiangsu, China) and measured at 405 nm by a microplate reader following the manufacturer's instructions.

### 3.3 Migration and invasion assays

The effect of menthol on A549 cell migration and invasion was further determined with Transwell chambers (Corning, NY, USA) without or with Matrigel (BD Biosciences, USA). Firstly, cells were pretreated with PBS or menthol (0.8 mM) and/or SC79 (4 μg/mL) for 24 h. Then cells were loaded in the Transwell migration chambers at a density of 1 × 10^5^. In the lower chambers, medium containing 10% FBS was added as chemoattractant. The chambers were incubated, and cell migration was allowed for 6 h. Then migratory cells on the lower surface were fixed by 4% paraformaldehyde after cells on the upper side of the membrane were removed. Cells were finally washed with PBS and stained with crystal violet and ethanol. The number of migratory cells was assessed by DMI3000B fluorescent microscope (Leica, Germany) at ×100 magnification.

### 3.4 Western blot analysis

After being seeded into 6‐well plates, cells were incubated with PBS and menthol (0.8mM) and/or SC79 (4 μg/mL) for 24 h. The total proteins were prepared by incubation with RIPA buffer with protease inhibitor and phosphatase inhibitor cocktail. The proteins were resolved in 12% SDS‐PAGE and subjected to immunoblotting analysis with antibodies specific for Caspase‐3, cleaved Caspase‐3, Akt, p‐Akt, Bcl‐2, Bax, MMP‐2, MMP‐9 and β‐actin (Cell Signaling Technology, USA). The western blots were quantified by ImageJ software.

### 3.5 In vivo studies

To further verify the anticancer activity of menthol, an in vivo model for tumorigenicity of A549 tumor cells was established in nude mice. Fifteen 5‐week‐old female nude mice weighting 20 g were purchased from Shanghai Laboratory Animal Company (SLAC, Shanghai). Cells (5 × 10^6^) suspended in Matrigel were subcutaneously (s.c.) injected into the nude mice. Three days later, mice bearing visible tumors averaged 6 mm^3^ in size were randomly divided into two groups: the PBS control group and menthol (20 mg/kg) group. Menthol dispersed in PBS was administered to the mice intraperitoneally (i.p.) every 2 days for totally 3 weeks. The animals were anesthetized with inhalation of 3% isoflurane and then sacrificed. Tumor masses were isolated, and tumor weight was measured.

### 3.6 Construction of an expression lentiviral vector for Akt

In the overexpression experiments, Akt pathway was PCR‐amplified and cloned at the AgeI site in the pGC‐FU lentiviral vector, which was digested by the restriction enzyme AgeI (NEB). The fragment of Akt was ligated with the pGC‐FU vector at the AgeI site. Successful cloning was verified by sequencing. According to the manufacturer's instructions, lentivirus vectors were produced by transient transfection into 293 T cells using calcium phosphate coprecipitation. The subsequent purification and concentration of virus were performed, respectively, using Millipore filtration and ultracentrifugation.

### 3.7 Statistical analysis

Statistical analysis was performed with GraphPad Prism software (version 7, San Diego, CA). One‐way ANOVA with Bonferroni's post hoc test (for equal variance) or Dunnett's T3 post hoc test (for unequal variance) was performed for comparisons among multiple groups. Statistically significant was considered when *p* < 0.05.

## 4 RESULTS

### 4.1 Menthol‐induced cytotoxicity and reduced AKT activity in A549 cells

**FIGURE 1:**
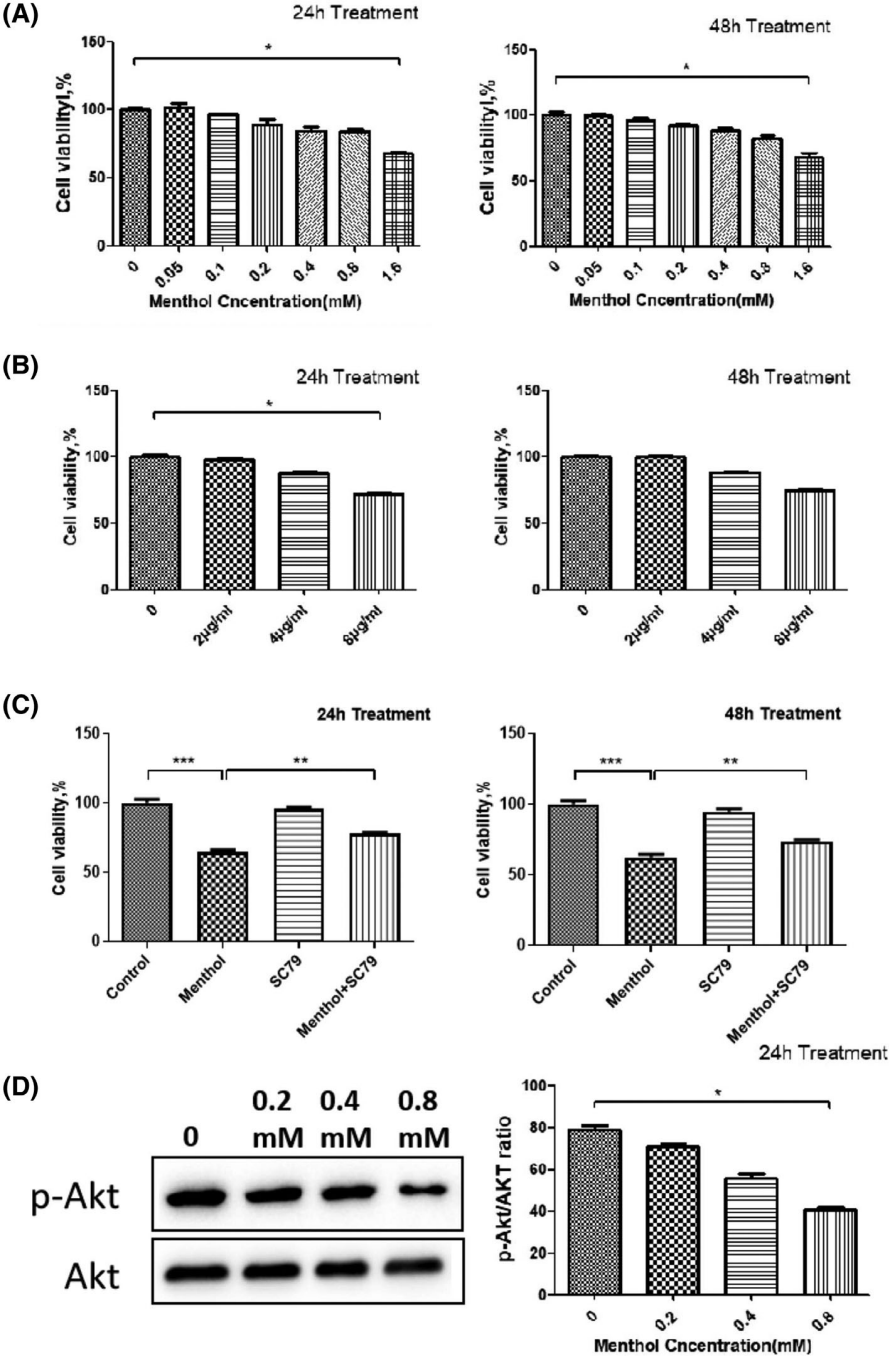
Cytotoxic effect of A549 cells by MTT assay. (A) After incubation of cells with indicated concentrations (0, 0.05, 0.1, 0.2, 0.4, and 0.8 mM) of menthol for 24 or 48 h, menthol exhibited a cytotoxic effect in a concentration‐dependent manner. (B) After cells were incubated with indicated concentrations (0, 2, 4, 8 μg/mL) of SC79 for 24 and 48 h, optimal concentration 4 μg/mL was chosen for subsequent experiment. (C) Menthol reduced A549 cell survival, but cotreatment of SC79 and menthol alleviated this cytotoxicity. (D) Menthol decreased the phosphorylation of Akt (p‐Akt) without changing total Akt. *P < 0.05.

To evaluate the antitumor activity of menthol in vitro, the cytotoxicity of menthol on A549 cells was assessed firstly. As shown in MTT assay, menthol exhibited cytotoxic effect on A549 cells in a concentration‐dependent manner both at 24 and 48 h time point (Figure 1A). Then the cytotoxicity of SC79, an agonist of Akt, on A549 cells was assessed. The optimal SC79 concentration of 4 μg/mL was chosen for subsequent experiment because of no significant alteration of cell survival (Figure 1B). Menthol treatment alone reduced the viability of A549 cells, but cotreatment of SC79 and menthol alleviated this cytotoxicity (Figure 1C). These results suggest that the antitumor effects of menthol may be influenced via Akt signaling pathway.

Akt signaling pathway has been demonstrated to function in regulating apoptosis and metastasis of tumor cells.^10^, ^11^, ^12^, ^13^ To explore the mechanism of inhibitory effect of menthol on A549 cells motility, the protein level and activated status of Akt signaling was detected by western blot assay afterwards. Figure 1D showed that menthol dramatically decreased the phosphorylation of Akt (p‐Akt) without changing the expression of total Akt.

### 4.2 Menthol promoted cell apoptosis of A549 cells

**FIGURE 2:**
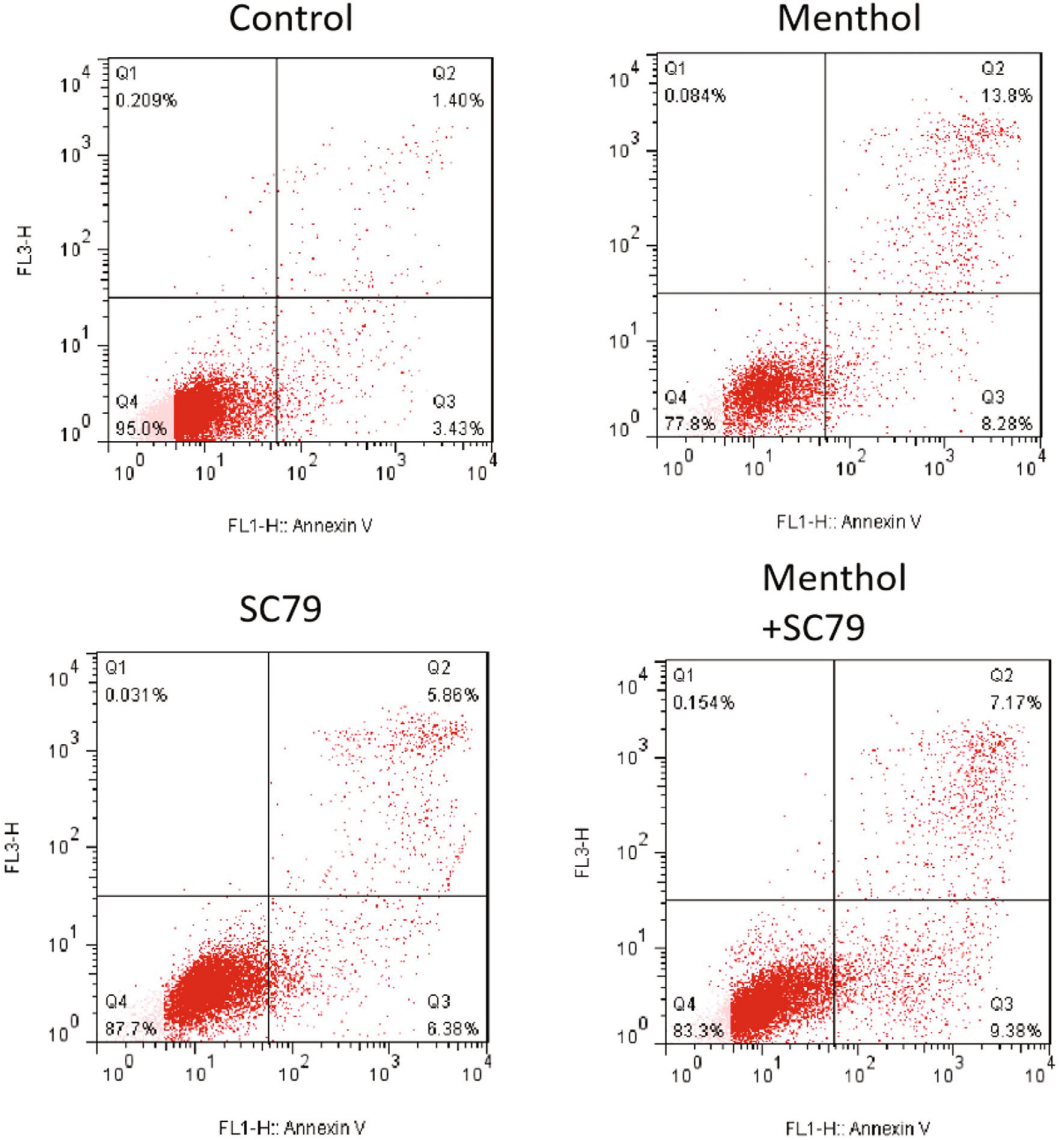
Menthol promoted apoptosis induction in A549 cells. Menthol increased the percentage of apoptotic cells and dead cells after incubation of 24 h (right upper panel). Cotreatment of SC79 decreased percentage of apoptotic or dead cells induced by menthol alone (right lower panel).

The apoptosis induction effect of menthol on A549 cells was investigated afterwards with Annexin V‐PI staining and flow cytometry. As shown in Figure 2, the percentage of apoptotic cells and dead cells increased after being treated with menthol for 24 h. However, the cotreatment of SC79 and menthol showed no significant increase of apoptotic or dead cells.

### 4.3 Menthol suppressed migration and invasion of A549 cells

**FIGURE 3:**
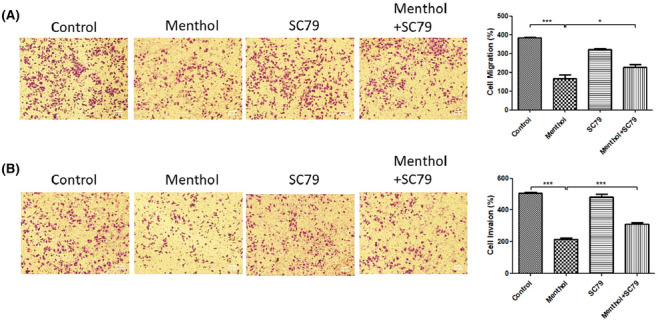
Effects of menthol on A549 cell migration and invasion. The migratory (A) and invasive (B) ability of A549 cells were hindered by menthol treatment, and the inhibiting effect was mitigated after cotreatment with SC79. *P < 0.05, ***P < 0.001.

The potential inhibitory function of menthol on migration and invasion of A549 tumor cells was performed by Transwell assay without or with Matrigel. The migratory (Figure 3A) and invasive (Figure 3B) ability of A549 cells was markedly inhibited by menthol treatment, but the inhibiting effect was mitigated after Akt activation by cotreatment with SC79.

### 4.4 Effects of menthol on the activity of Caspase‐3

**FIGURE 4:**
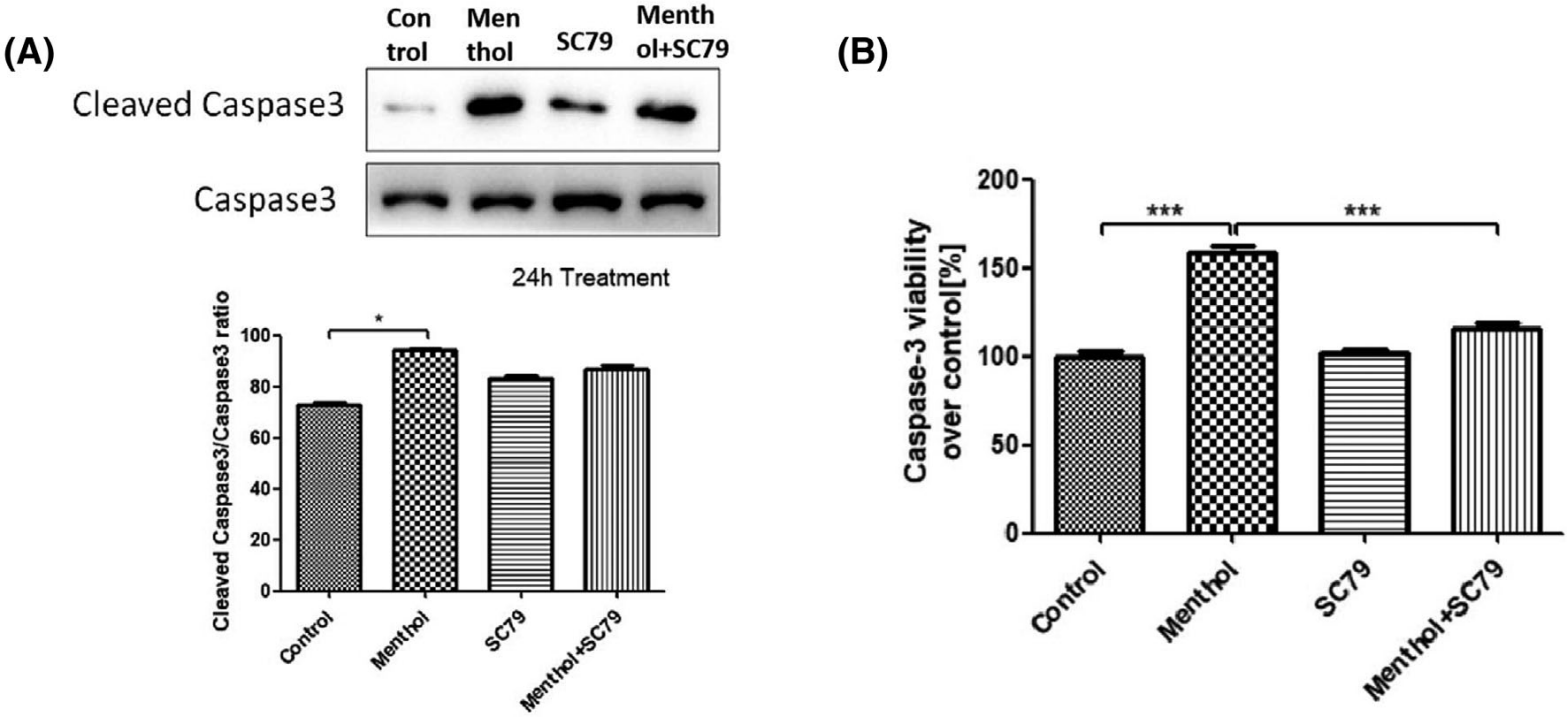
Caspase‐3 activity was measured in A549 cells. Menthol increased expression level of cleaved Caspase‐3 (A) and Caspase‐3 activity (B), and the effect of menthol was impeded by Akt activation with SC79. *P < 0.05, ***P < 0.001.

The level of Caspase‐3 in A549 cells was analyzed by testing the protein expression and activation of Caspase‐3. Presented in Figure 4A,B, the expression level of cleaved Caspase‐3 increased after cells were exposed to menthol, indicating that Caspase‐3 activation was also involved in menthol‐induced A549 cell apoptosis. The apoptosis‐induction effect of menthol was impeded by Akt activation with SC79.

### 4.5 Effects of menthol on apoptosis‐ and metastasis‐related protein expression of A549 cells

**FIGURE 5:**
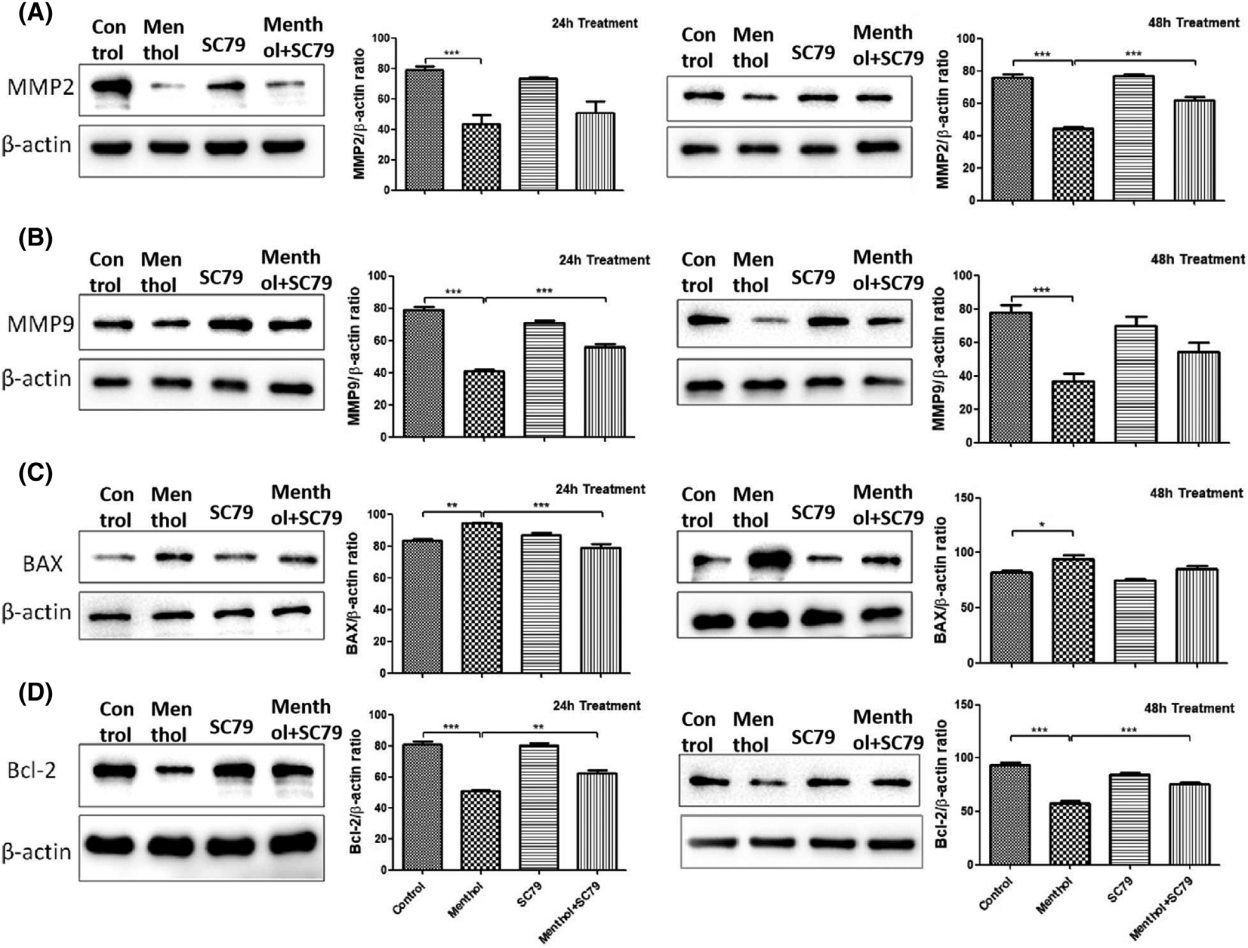
Western blot analysis after menthol and SC79 treatment. Menthol decreased the expression of MMP‐2 (A), MMP‐9 (B), and Bcl‐2 (D) while increased Bax (C) protein level compared with the control group. Akt activation by SC79 inhibited the toxic effects of menthol. *P < 0.05, **P < 0.01, ***P < 0.001.

Results of the western blots were shown in Figure 5. Menthol treatment significantly changed the expression level of apoptosis‐related proteins. After menthol treatment, the expression of Bax increased, while the Bcl‐2 protein expression decreased compared with the control group. The cotreatment of menthol and SC79 mitigated the Bax expression increase at 24 h (Figure 5C) and Bcl‐2 decrease at 24 and 48 h (Figure 5D) caused by menthol alone.

As MMPs play important roles in promoting cancer invasion and metastasis by digesting the extracellular matrix (ECM) and destroying basement membrane,^14^ the protein expression of MMPs was detected to explore whether the inhibited motility of A549 cells by menthol was regulated by MMPs. As in Figure 5A,B, MMP‐2 and MMP‐9 showed significant reduction with the administration of menthol. Activating Akt with SC79 mitigated MMP‐2 decrease at 48 h and MMP‐9 decrease at 24 h.

### 4.6 Tumor‐suppressing activity of menthol in vivo

**FIGURE 6:**
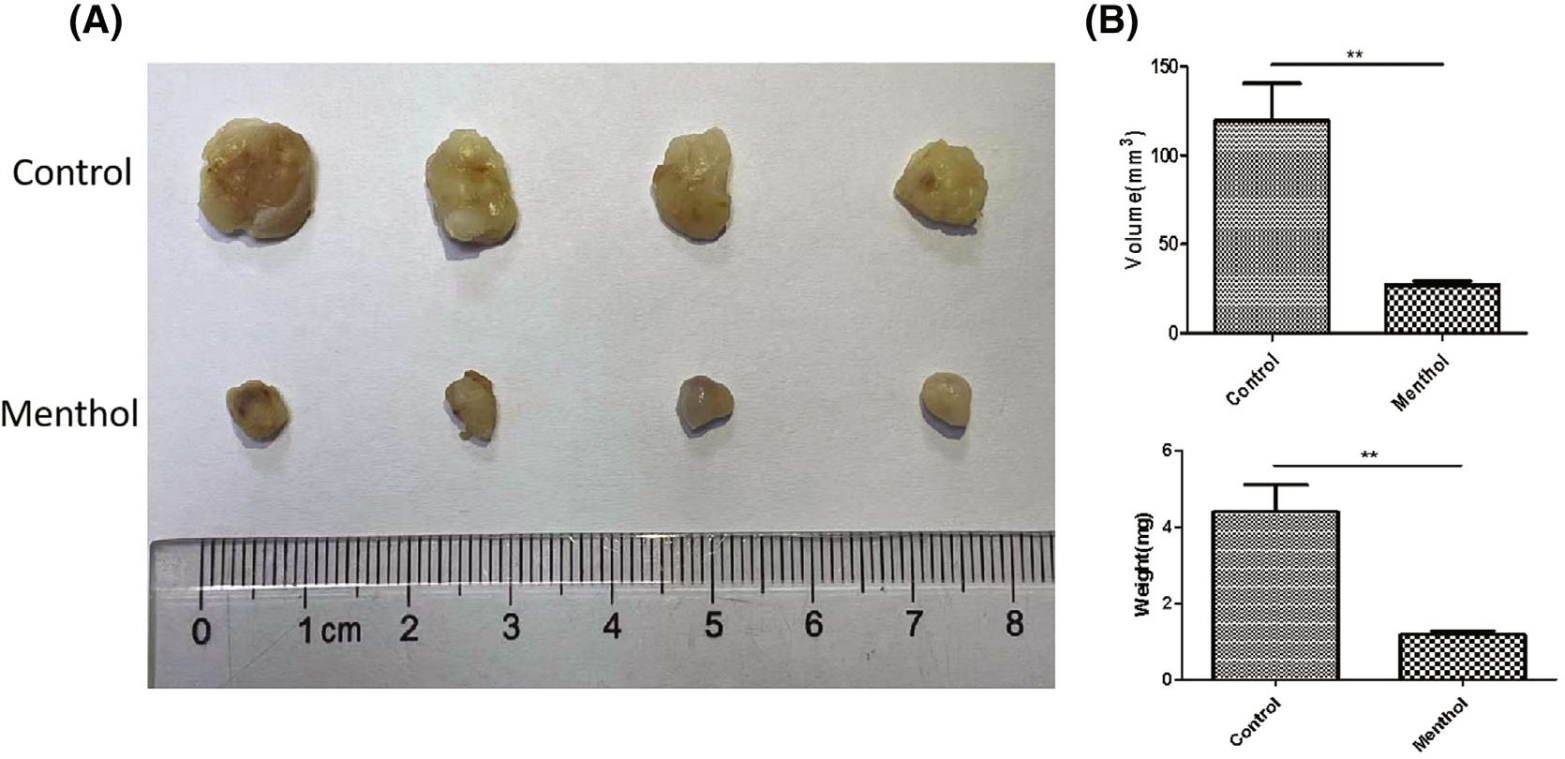
Antitumor effect of menthol in vivo. The tumor size significantly decreased by menthol administration compared with the control (A). The volume and weight of tumors were analyzed (B). **P < 0.01.

Figure 6 presented tumor images in each group. As anticipated, the tumor size significantly decreased with 20 mg/kg menthol treatment compared with the control.

### 4.7 Menthol‐induced cytotoxicity in Akt over‐expressed A549 cells

We successfully constructed Akt over‐expressed A549 cells (supporting information Figure S1A). As shown in MTT assays, after cells were treated with menthol for 24 or 48 h, menthol exhibited a cytotoxic effect on A549 cells (supporting information Figure S1B). The menthol treatment reduced the viability of A549, but this effect was hindered by Akt over‐expression, both at 24 and 48 h.

### 4.8 Effects of menthol on Caspase‐3 activity in Akt over‐expressed A549 cells

As demonstrated in supporting information Figure S2A,B, the expression level of cleaved Caspase‐3 increased after cells were exposed to menthol, which means Caspase‐3 activation was also involved in menthol‐induced apoptosis. When the Akt was over‐expressed in A549 cells, the effect of menthol on Caspase‐3 activity was weakened.

### 4.9 Menthol suppressed motility of A549 cells over‐expressing Akt

As presented in the supporting information Figure S3A,B, the migratory and invasive capability of A549 cells was repressed by treatment of menthol. The motility of tumor cells was markedly inhibited by menthol after 24 h, but the inhibiting effect was decreased in Akt over‐expressed A549 cells.

### 4.10 Effects of menthol on apoptosis‐ and metastasis‐related protein expression in Akt over‐expressed A549 cells

In the supporting information Figure S4, menthol treatment significantly changed the expression level of apoptosis‐related proteins, both at 24 h and 48 h. After menthol treatment, the expression of Bax increased while the Bcl‐2 protein level decreased compared with the control group, and MMP‐2 and MMP‐9 protein showed significantly decrease after treatment with menthol. However, after menthol treatment in Akt over‐expressed A549 cells, the changes of Bcl‐2, Bax and MMPs proteins level were mitigated, compared with menthol‐treated vehicle group.

### 4.11 Effects of menthol on Akt over‐expressed H1299 cells

Furthermore, we constructed Akt over‐expressed H1299 cells to verify our results (supporting information Figure S5A). After cells were treated with menthol for 24 h and tested by MTT, menthol exhibited a cytotoxic effect on H1299 cells (supporting information Figure S5B). The menthol treatment reduced the viability of cells, but this effect was hindered by Akt over‐expression. In addition, menthol treatment significantly increased the level of cleaved Caspase‐3 in H1299 cells, but when the Akt was overexpressed in cells, the increased expression level of cleaved Caspase‐3 fell after cells was exposed to menthol.

On the other hand, menthol treatment significantly changed the expression level of apoptosis‐related proteins. After menthol treatment, compared with the control group, the expression of Bax increased, while the Bcl‐2 protein level, as well as MMP‐2 and MMP‐9 protein, significantly decreased after treatment with menthol. However, in menthol treatment, Akt overexpressed H1299 cells, the changes of apoptosis‐related proteins level were alleviated compared with menthol‐treated vehicle group.

## 5 DISCUSSION

Menthol has been previously demonstrated to exhibit an anticancer activity in several tumor cell lines.^7^, ^8^, ^15^ The present study preliminarily elucidated that menthol suppressed A549 cells through Akt signaling pathway.

Apoptosis is not only necessary in maintaining homeostasis of cell growth but also plays an important role in preventing tumor progress.^16^, ^17^ As important regulators of cell apoptosis, the Bcl‐2 protein family consists of pro‐apoptotic Bad and Bax and anti‐apoptotic members including Bcl‐2.^18^ As a crucial pro‐apoptotic Bcl‐2‐family protein, Bax exists in the cytosol. It translocates to mitochondria on the induction of apoptosis.^19^, ^20^, ^21^, ^22^ Bax is capable of activating caspases,^23^, ^24^ which then lead to apoptosis by cleavage of key substrates.^25^, ^26^, ^27^, ^28^ Besides, upregulation of Bcl‐2 has been identified as a critical mechanism by which cell survival is promoted.^29^, ^30^, ^31^, ^32^ In the present study, western blot analysis showed menthol promoted A549 cell apoptosis by increasing Bax level, activation of Casepase‐3, and decreasing Bcl‐2 expression.

Akt has been demonstrated as a key regulator of apoptosis.^11^, ^12^ Akt promotes cell survival through upregulation of Bcl‐2 by transcriptional activation.^33^ Activated Akt (phosphorylated Akt, p‐Akt) phosphorylates Bad protein to the nonactivated state and induces the subsequent reduction of its downstream Bax, thus exhibiting anti‐apoptosis effect.^34^, ^35^ Furthermore, Caspase‐3 activation can be blocked, and cells are rescued from apoptotic death by activated Akt.^36^, ^37^, ^38^ As anticipated in this study, p‐Akt was decreased in menthol‐treated A549 cells with the total Akt protein level unchanged. These results suggested that Akt pathway might, in part, be involved in menthol‐induced apoptosis.

The migration or invasion of cancer cells represents important characteristic of tumor metastasis. The MMPs protein family plays a central role in malignant metastasis. MMPs can digest basement membranes and components of the ECM; thus, cancer cells can penetrate into the bloodstream.^39^, ^40^ As documented in many researches, both MMP‐2 and MMP‐9 proteins are abundantly expressed in different malignancies and these proteins facilitate cancer metastasis.^41^, ^42^, ^43^ Cells need the adhesive contacts and cellular networks for adhering to the basement membrane, but these networks can be cleaved by MMP‐2 so that cancer cell motility is promoted.^42^, ^44^, ^45^ Downregulation of MMP‐2 hinders tumor cell invasion.^46^, ^47^ MMP‐9 was also demonstrated to promote cell invasion and metastasis of Lewis lung carcinoma.^43^

The expression of MMPs can be induced by Akt‐mediated cell signals.^13^, ^14^, ^48^ It was reported by Guo et al.^49^ that activating the PI3K/Akt signaling pathway upregulates MMP‐9. Xu et al.^50^ also found that in normal fibroblasts, the MMP‐9 expression can be enhanced by the PI3K/Akt pathway activation. Our results indicated that menthol inhibited A549 cell invasion by decreasing the expression of MMP‐2, MMP‐9, and p‐Akt. It can be speculated that menthol inhibited migration and invasion through the inactivation of Akt and subsequent downregulation of MMPs protein expression.

At the same time, there are limitations in the present study. Only two NSCLC cell lines were utilized, and some of the upstream and downstream proteins of Akt signaling pathway were not detected. Further in‐depth investigations will be needed in the future.

## 6 CONCLUSION

In conclusion, the current study demonstrated for the first time that menthol was capable of inhibiting NSCLC A549 and H1299 cells, both in vitro and in vivo. Menthol promoted apoptosis and suppressed migratory and invasive capability of A549 cells with up‐regulating Bax and cleaved Caspase‐3, along with downregulation of Bcl‐2, MMP‐2, and MMP‐9. The inhibitory effect of menthol on A549 cells might through Akt signaling pathway, and menthol might be able to play a role in the therapy of NSCLC.

## AUTHOR CONTRIBUTIONS

Conception and design: Zhiyu Liu. Administrative support: Zhiyu Liu. Provision of study materials or patients: Chunlin Li. Collection and assembly of data: Haiyang Hu. Data analysis and interpretation: Xiong Qin. Manuscript writing: All authors. Final approval of manuscript: All authors.

## CONFLICT OF INTEREST STATEMENT

The authors declare no competing interests.

## ETHICS STATEMENT

All procedures were conducted in accordance with the National Institutes of Health guide for the care and use of Laboratory animals and conformed to our institutional ethical guidelines for animal experiments (Permit Number: SYXK 2019‐0028).

## Supporting information


**Figure S1.** Cytotoxic effect of Akt over‐expressed A549 cells by MTT assay. The expression of Akt was shown in Figure 1A. After cells were incubated with menthol for 24 h or 48 h (B), menthol exhibited no cytotoxic effect on Akt over‐expressed A549 cells. ***P < 0.001.

**Figure S2.** Cleaved Caspase‐3 was measured in Akt over‐expressed A549 cells. Vehicle cells and Akt over‐expressed cells were treated with menthol for 24 h and subjected to western blot (A) and Caspase‐3 Activity Assay Kit (B) analysis with specific antibodies. **P < 0.01, ***P < 0.001.

**Figure S3.** Effects of menthol on Akt over‐expressed A549 cell migration and invasion. Medium containing 10% FBS was placed in the lower chambers as chemoattractant and that with no FBS was in the upper. The migratory cells (A) and invaded cells (B) on the lower side were fixed, stained and counted by fluorescence microscopy. **P < 0.01, ***P < 0.001.

**Figure S4.** Western blot analysis of apoptosis‐ and metastasis‐ related proteins. Vehicle cells and Akt over‐expressed cells were treated with menthol for 24 h or 48 h and subjected to western blot analysis with specific antibodies. MMP‐2 (A), MMP‐9 (B), Bax (C) and Bcl‐2 (D) were presented and β‐actin served as an internal control. ***P < 0.001.

**Figure S5.** The expression of Akt was shown in Figure 1A. Cytotoxic effect of Akt over‐expressed H1299 cells by MTT assay, after cells were incubated with menthol for 24 h (B). Cleaved Caspase‐3 was measured in Akt over‐expressed H1299 cells. Vehicle cells and Akt over‐expressed cells were treated with menthol for 24 h and subjected to western blot and Caspase‐3 Activity Assay Kit (C) analysis with specific antibodies. Vehicle cells and Akt over‐expressed cells were treated with menthol for 24 h and subjected to western blot analysis with specific antibodies. MMP‐2, MMP‐9, Bax and Bcl‐2 (D) were presented and β‐actin served as an internal control. **P < 0.01, ***P < 0.001, ****P < 0.0001.

## Data Availability

The datasets used and/or analyzed during the current study are available from the corresponding author on reasonable request.
