# Supplementary figures and images for: Menthol induces apoptosis and inhibits proliferation and migration of nonsmall cell lung carcinoma in vitro and in vivo through Akt pathway
Source: Clin Respir J. 2023 Nov 27;17(12):1265–75. doi: 10.1111/crj.13713 (PMC10730467; doi:10.1111/crj.13713)

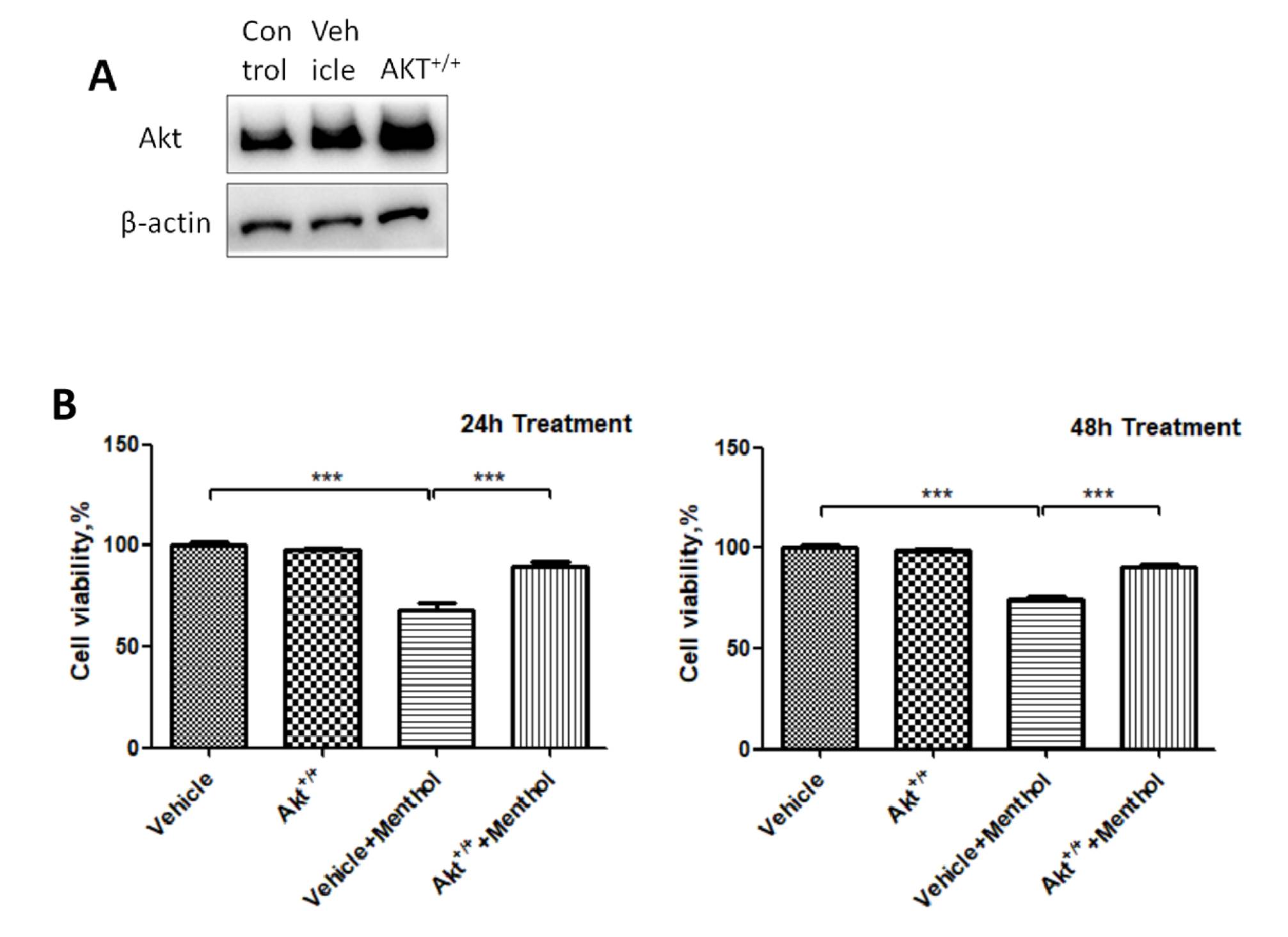

Supplement: Supplementary file 1 — Figure S1. Cytotoxic effect of Akt over‐expressed A549 cells by MTT assay. The expression of Akt was shown in Figure 1A. After cells were incubated with menthol for 24 h or 48 h (B), menthol exhibited no cytotoxic effect on Akt over‐expressed A549 cells. ***P < 0.001. [file CRJ-17-1265-s001.jpg]

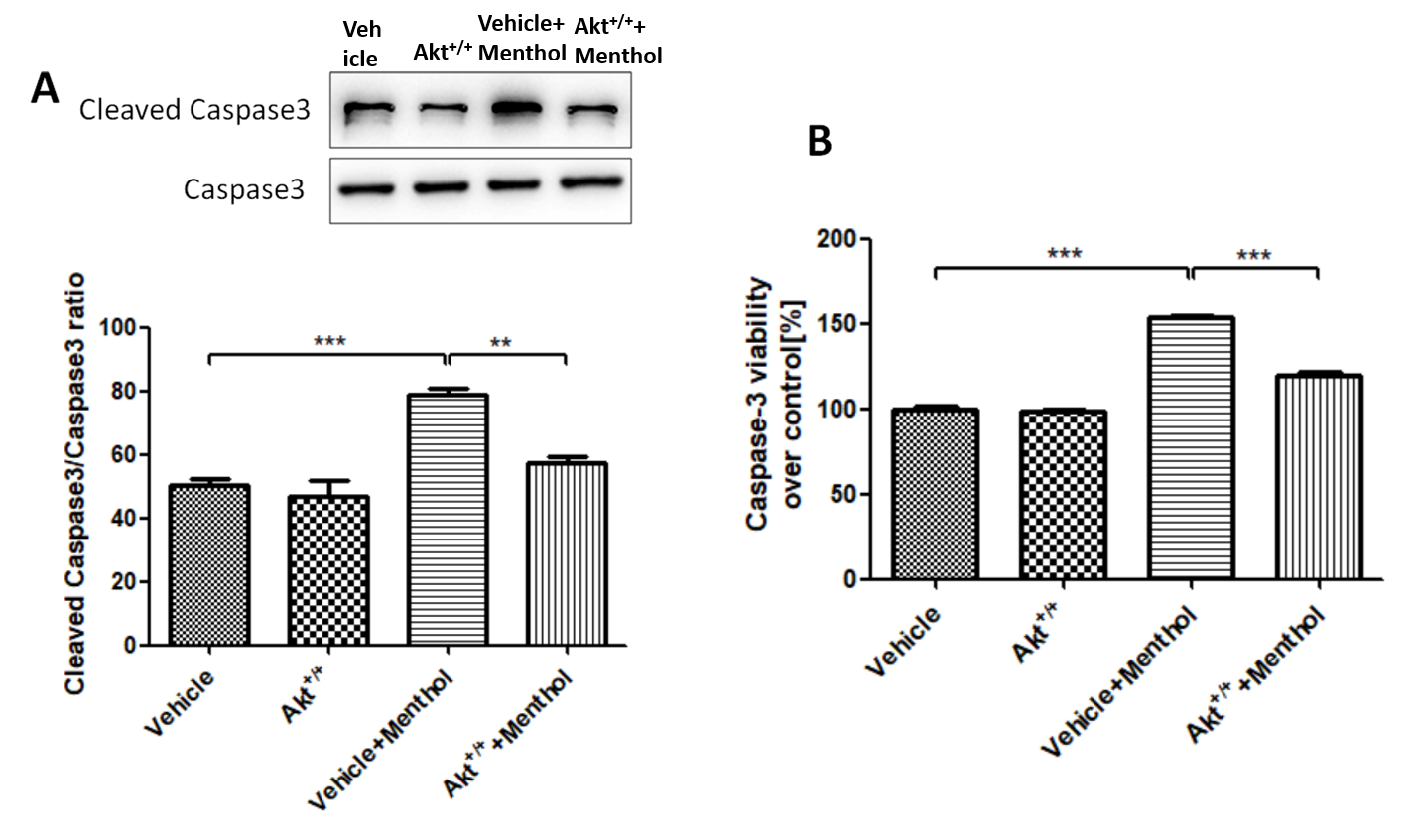

Supplement: Supplementary file 2 — Figure S2. Cleaved Caspase‐3 was measured in Akt over‐expressed A549 cells. Vehicle cells and Akt over‐expressed cells were treated with menthol for 24 h and subjected to western blot (A) and Caspase‐3 Activity Assay Kit (B) analysis with specific antibodies. **P < 0.01, ***P < 0.001. [file CRJ-17-1265-s004.png]

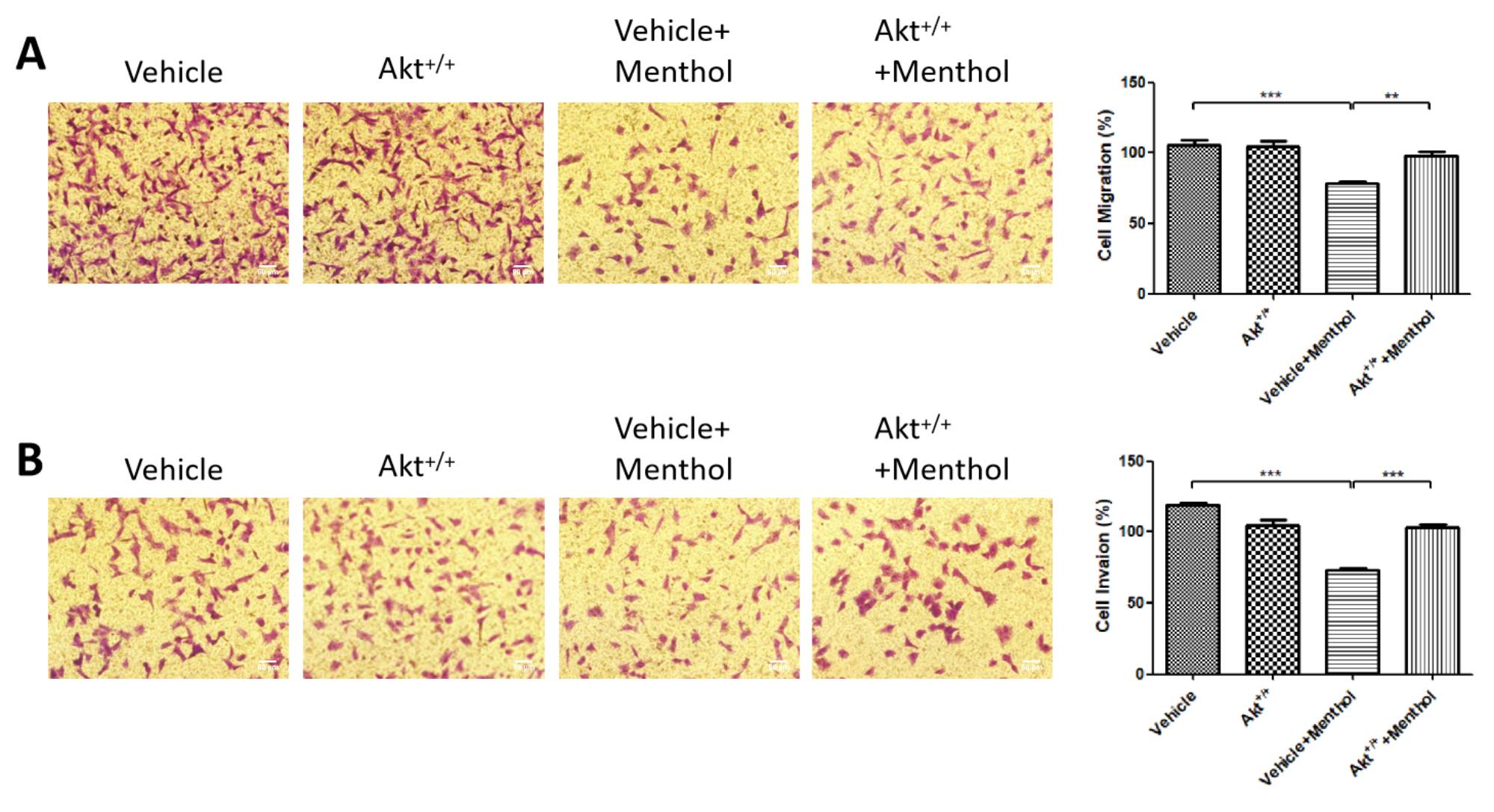

Supplement: Supplementary file 3 — Figure S3. Effects of menthol on Akt over‐expressed A549 cell migration and invasion. Medium containing 10% FBS was placed in the lower chambers as chemoattractant and that with no FBS was in the upper. The migratory cells (A) and invaded cells (B) on the lower side were fixed, stained and counted by fluorescence microscopy. **P < 0.01, ***P < 0.001. [file CRJ-17-1265-s003.jpg]

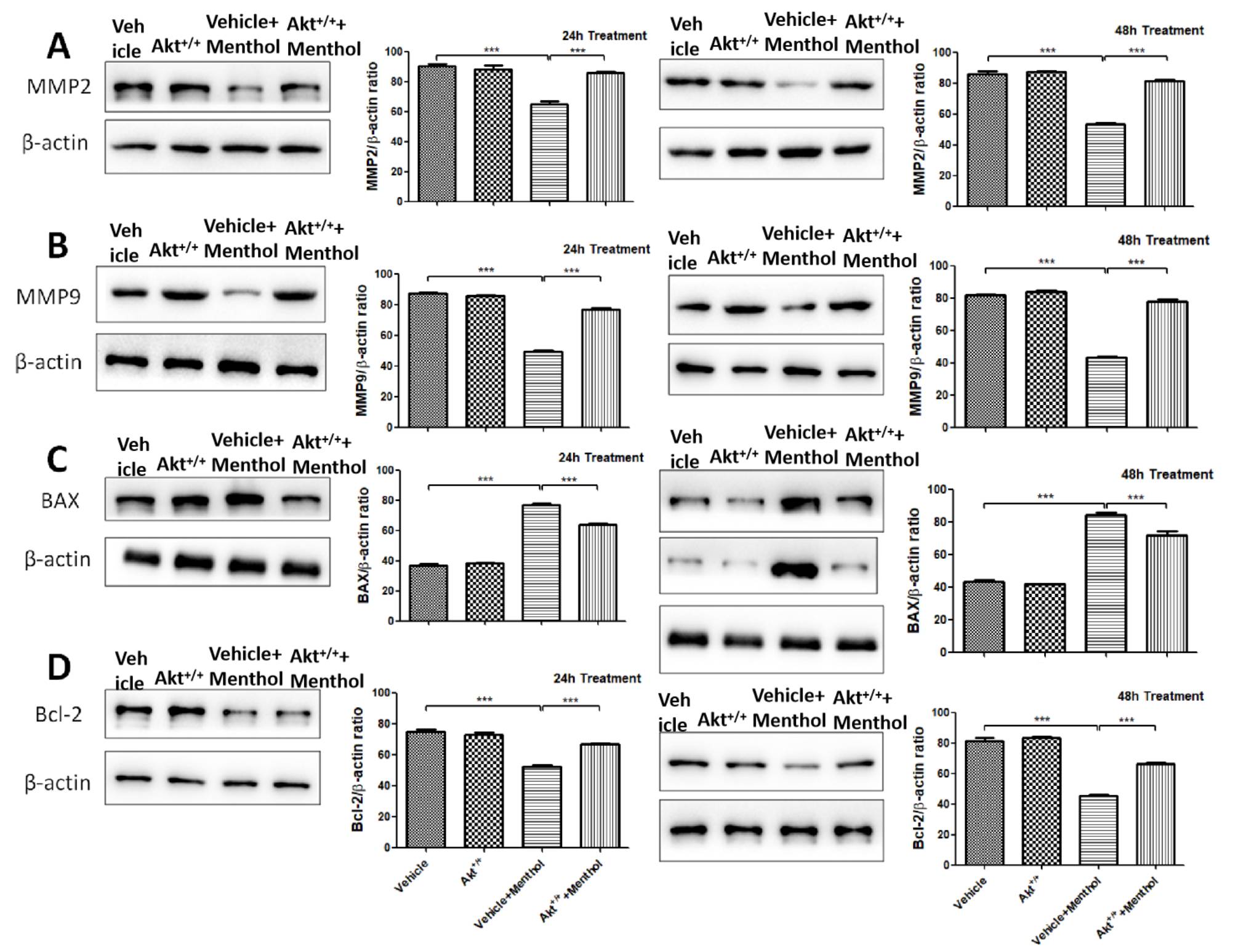

Supplement: Supplementary file 4 — Figure S4. Western blot analysis of apoptosis‐ and metastasis‐ related proteins. Vehicle cells and Akt over‐expressed cells were treated with menthol for 24 h or 48 h and subjected to western blot analysis with specific antibodies. MMP‐2 (A), MMP‐9 (B), Bax (C) and Bcl‐2 (D) were presented and β‐actin served as an internal control. ***P < 0.001. [file CRJ-17-1265-s002.jpg]

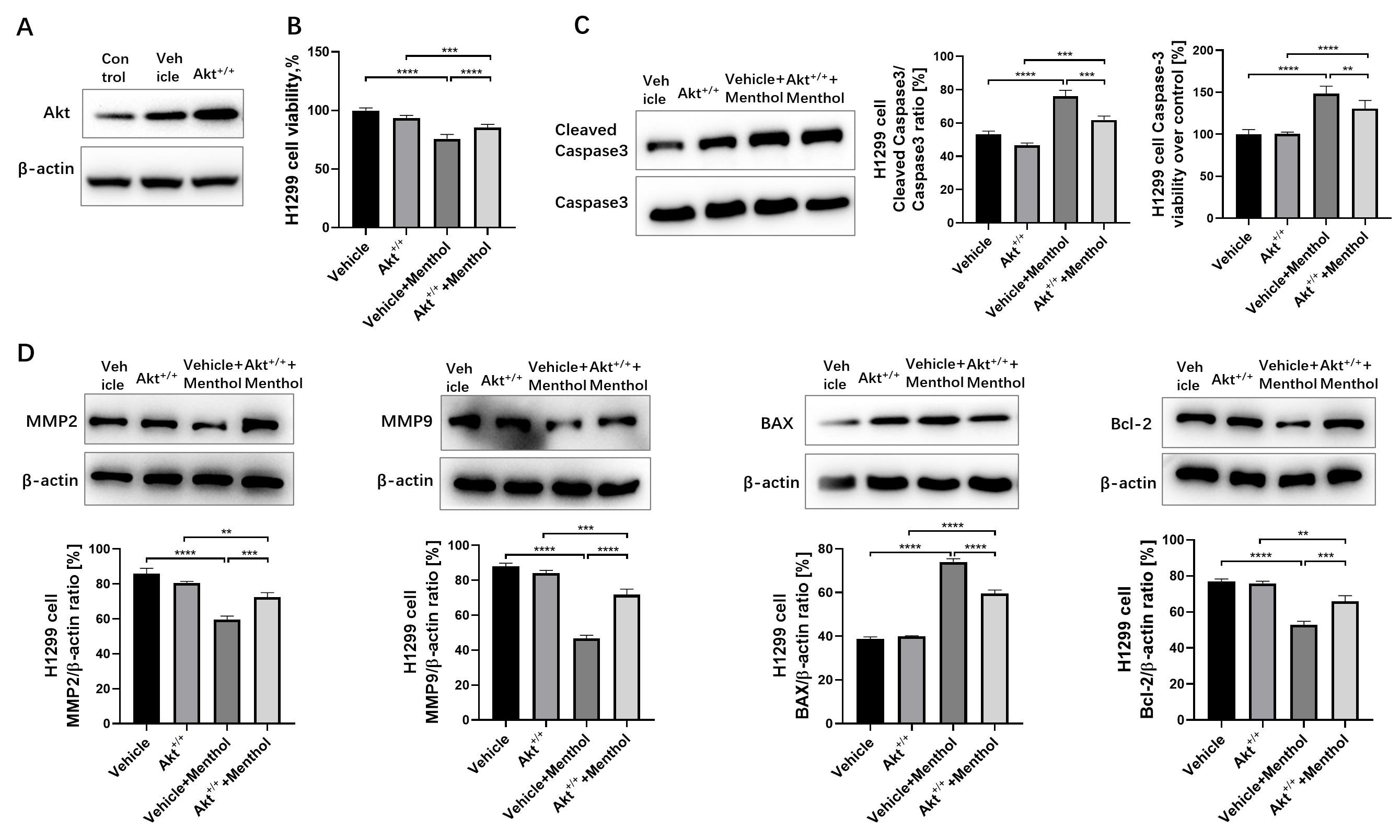

Supplement: Supplementary file 5 — Figure S5. The expression of Akt was shown in Figure 1A. Cytotoxic effect of Akt over‐expressed H1299 cells by MTT assay, after cells were incubated with menthol for 24 h (B). Cleaved Caspase‐3 was measured in Akt over‐expressed H1299 cells. Vehicle cells and Akt over‐expressed cells were treated with menthol for 24 h and subjected to western blot and Caspase‐3 Activity Assay Kit (C) analysis with specific antibodies. Vehicle cells and Akt over‐expressed cells were treated with menthol for 24 h and subjected to western blot analysis with specific antibodies. MMP‐2, MMP‐9, Bax and Bcl‐2 (D) were presented and β‐actin served as an internal control. **P < 0.01, ***P < 0.001, ****P < 0.0001. [file CRJ-17-1265-s005.png]
